# Impact of implant thread design on insertion torque and osseointegration: a preclinical model

**DOI:** 10.4317/medoral.25576

**Published:** 2022-09-29

**Authors:** Ernesto B Benalcázar-Jalkh, Vasudev Vivekanand Nayak, Christina Gory, Andres Marquez-Guzman, Edmara TP Bergamo, Nick Tovar, Paulo G Coelho, Estevam A Bonfante, Lukasz Witek

**Affiliations:** 1 Biomaterials Division, New York University College of Dentistry, New York, NY USA; 2 Department of Prosthodontics and Periodontology, University of São Paulo - Bauru School of Dentistry, Bauru, SP, Brazil; 3 Department of Mechanical and Aerospace Engineering, New York University Tandon School of Engineering, Brooklyn, NY, USA; 4 Department of Oral and Maxillofacial Surgery, New York University, Langone Medical Center and Bellevue Hospital Center, New York, NY, USA; 5 Department of Surgery, University of Miami Miller School of Medicine, Miami, FL, USA; 6 Department of Biomedical Engineering, New York University Tandon School of Engineering, Brooklyn, NY, USA

## Abstract

**Background:**

Successful osseointegration of endosteal dental implants has been attributed to implant design, including the macro-, micro- and nano- geometric properties. Based on current literature pertaining to implant design, the resultant cellular and bone healing response is unknown when the thread thickness of the implants is increased, resulting in an increased contact area in implants designed with healing chambers. The aim of this study was to evaluate the effect of two implant designs with different thread profiles on the osseointegration parameters and implant stability at 3- and 6-weeks *in vivo* using a well-established preclinical dog model.

**Material and Methods:**

A total of 48 type V Ti alloy implants were divided in two groups according to their thread design (D1= +0.1x/mm and D2= +0.15x/mm) and placed in an interpolated fashion into the radii of six beagles. Insertion torque was measured at time of placement, radii were extracted for histological processing following 3- and 6-week healing intervals. Histologic and histomorphometric analyses were performed in terms of bone to implant contact (%BIC) and bone area fraction occupancy within implant threads (%BAFO). Statistical analyses were performed through a linear mixed model with fixed factors of time and implant thread design.

**Results:**

Surface roughness analysis demonstrated no significant differences in Sa and Sq between D1 and D2 implant designs, which confirmed that both implant designs were homogenous except for their respective thread profiles. For insertion torque, statistically significant lower values were recorded for D1 in comparison to D2 (59.6 ± 11.1 and 78.9 ± 10.1 N⋅cm, respectively). Furthermore, there were no significant differences with respect to histological analysis and histomorphometric parameters, between D1 and D2 at both time points.

**Conclusions:**

Both thread profiles presented equivalent potential to successfully osseointegrate in the osteotomies, with D2 yielding higher mechanical retention upon placement without detrimental bone resorption.

** Key words:**Thread design, dental implants, osseointegration, implant stability, microgeometry.

## Introduction

The successful osseointegration of endosteal dental implants is established on the effective recruitment of osteogenic cells during the healing process. This is dependent on a variety of factors, including the macro-, micro- and nano- geometric properties of implant design along with its interplay with the surgical preparation/technique (1,2). Upon implant placement, the establishment of primary stability has been considered one of the fundamental criteria influencing implant success (3). The mechanical interlock between the native bone and the device is meant to prevent micromotions that can disrupt the healing process ultimately impeding the establishment of secondary stability (4). Traditionally, primary stability is associated with high insertion torque values, obtained through undersized osteotomy preparations. However, such approach results in strain and microcrack formation in the bone, which may lead to compression necrosis and subsequently to bone remodeling, increasing osseointegration times (5).

Previous literature have demonstrated that small design variations may positively influence implant stability and potentially bone integrity (6). Modifications in thread-width, thickness and pitch have been evaluated, aiming to enhance primary stability (6-8). However, when implant stability is primarily dependent on implant design and/or undersized osteotomies; the strain and subsequent interfacial remodeling of bone is unavoidable (9). Therefore, the interplay between macrogeometric design parameters and osteotomy preparation is fundamental to enhance primary stability while promoting the efficient achievement of complete osseointegration (10,11). Implants designed to interact with the osteotomy preparation and allow for the formation of healing chambers between the threads, implant inner diameter, and the osteotomy walls have been suggested to avoid a purely interfacial remodeling osseointegration (1,9,10,12-15). In this scenario, primary stability is achieved by the frictional interaction of the outer regions of the threads that engage bone and allow for a hybrid healing osseointegration pathway, where interfacial remodeling and intramembranous-like osseointegration take place at the outer portion of the threads and within the healing chambers, respectively (16). While this conFiguration seems to promote favorable results to hasten osseointegration, different macrogeometric modifications that may further enhance primary stability in this particular scenario have not been fully explored in previous literature.

For example, the modification of the thread profile may affect the potential contact area between the implant and the native bone at the outer aspect of the implant threads (17). If the bone to implant contact area is increased, an increase in primary stability may be expected. However, if the resultant stresses are too high, osteogenic cellular remodeling can lead to bone resorption, decrease in implant stability, and subsequent clinical failure (9). Therefore, modifications to the macro-geometry that intend to improve primary stability must aim to limit compressive stress and to preserve the normal bone healing pattern, which is essential for the long-term osteointegration (1,18). Based on current literature, the resultant cellular and bone healing response is unknown when the thread thickness of the implants is increased, resulting in an increased contact area in implants designed with healing chambers. Therefore, the current *in vivo* study aimed to evaluate the effect of two different thread designs, D1 and D2, on the insertion torque and osseointegration parameters of titanium implants with healing chambers in a canine model. The postulated hypothesis of the present study was that the increase of the implant contact area produced by the thread design of D2 will significantly affect the maximum insertion torque values with no detrimental effect on the osseointegration parameters and healing process.

## Material and Methods

- Implant design and surface characterization

Forty-eight-screw root form type V Ti alloy implants with 4 mm diameter and 10 mm length (Colosso, Emfils, Itu, Brazil) were used for this study. Implants were divided in two groups according to thread profiles, D1 and D2. For both groups, thread sizes were gradually increased cervically starting from the apex. D1 was given a thread size of 0.5x apically and 1.5x cervically (IR = +0.1x/mm), and D2 was designed with a 0.5x thread size apically and 2.0x cervically (IR = +0.15x/mm). All implants received surface treatment by micro blasting with 100 µm particles at 100 m/s with a surface coverage of 5 g/in2 (C3 Microblasted, Comco, INC, Burbank, CA).

Surface characterization was performed via scanning electron microscopy (SEM) (HITACHI S-3500N, Hitachi Science Systems, Ltd., Japan), at various magnifications under an acceleration voltage of 15 kV and WD from 6.7 – 8.4 mm. Surface roughness parameters were evaluated at the flat region of the implant cutting edges using optical interferometry (IFM) (Phase View 2.6, Palaiseau, France), where twelve measurements were performed in four samples of each group. Sa (arithmetic average high deviation) and Sq (root mean square) surface roughness parameters were determined using a Gaussian filter size of 100 μm × 100 μm to remove errors of form and waviness.

- Preclinical In Vivo Model

Following the approval of the Ethics Committee for Animal Research at Ecole Nacionale Veterinaire Maíson Alfort (ENVA, Maíson Alfort, France) six adult male beagle dogs (~1.5 years old) were acquired and allowed to acclimate for one week prior to surgical procedures. For all surgical procedures, intramuscular atropine sulfate (0.044 mg/kg) and xylazine chlorate (8 mg/kg) were delivered prior to general anesthesia to lower the parasympathetic activity of muscles and to induce the following anesthesia. A 15 mg/kg ketamine chlorate dose was administered to reach general anesthesia.

After shaving of the hair and antiseptic cleaning with iodine solution, the central region of the radius diaphysis was exposed for implant placement. Four implants were placed along the radius from proximal to distal in an interpolated fashion, between radii to minimize bias (sites 1 to 4 from proximal to distal). The implants remained *in vivo* for 3 and 6 weeks (right and left radii provided samples that remained *in vivo* for 3 and 6 weeks, respectively). Upon insertion, the maximum insertion torque was recorded using a digital torque meter (Tohnichi, Tokyo, Japan) equipped with a 200 N⋅cm load cell for each implant. To avoid tissue overgrowth, each implant also was given a cover screw. Soft tissue was sutured in layers according to standard procedures, with the periosteum sutured with Vicryl 4-0 (Ethicon, Johnson & Johnson, Miami, FL) and the skin with 4-0 nylon (Ethicon).

Following implant placement, each dog received a single dose of benzyl penicillin benzatine (20,000 Ui/kg) intramuscularly and a ketoprofen 1% (1 mL/5 kg) as postoperative antibiotics and anti-inflammatory medication. Six weeks after the first surgical procedure, euthanasia was performed by an anesthesia overdose. At necropsy, the limbs were retrieved by sharp dissection, the soft tissue was removed using surgical blades, and an initial clinical evaluation was performed to confirm implant stability.

- Histomorphometric and histologic evaluations

The *en bloc* samples were cut into blocks and immersed in 10% buffered formalin solution for 24 hours. The blocks were then submitted to a step-by-step dehydration protocol in ethanol solutions ranging from 70% to 100%. Once dehydrated, the samples were infiltrated and embedded in a methacrylate-based resin (Technovit 9100, Heraus Kulzer GmbH, Wehrheim, Germany) according to the manufacturer’s instructions. The embedded samples were then cut into slices of about 300 µm thick, aiming the center of the implant along its long axis, with a precision diamond saw (Isomet 2000, Beuhler, Lake, Bluff, IL), and glued to acrylic plates with an acrylate-based cement. Slides were allowed to set for 24-hours before grinding and polishing ensued. Sections were then reduced to a final thickness of ~100 µm using a series of SiC abrasive papers (400, 600, 800 and 1200 grit) with a grinding/polishing machine (Metaserv 3000, Beujler) under water irrigation. The sections were then stained in a two-step process using Stevenel’s Blue and Van Gieson’s. Slides were scanned using an Aperio Scanscope (ScanScope GL, Vista, CA, USA) then referred to optical microscopy at 50x to 200x magnification (Leica DM2500M, Leica Microsystems, GmbH, Wetzlar, Germany) for histologic and histomorphometric analysis. Qualitative and quantitative histological analyses were performed using image analysis software (ImageJ, NIH, Bethesda, MD). Bone-to-implant contact (BIC) and bone area fraction occupancy (BAFO) were performed to evaluate the osseointegration parameters around the complete implant’s surface and within the thread area, respectively. All quantitative analyses were performed by a calibrated single blind evaluator after a good intraclass correlation coefficient (between 0.9 to 1) was obtained in the intra-rater reliability measurements.

- Statistical Analysis

The results from insertion torque test and histomorphometrical analyses (BIC and BAFO) are presented as mean values with corresponding 95% confidence interval (mean ± 95% CI). Prior to statistical analysis, data were submitted to the Shapiro – Wilk test, where normal distribution was observed, followed by statistical analysis using a general linear mixed model analysis (IBM SPSS v23 software, IBM Corp., Armonk, NY). The histomorphometry results (BIC and BAFO) along with the torque out comparisons were evaluated as function of thread design and time *in vivo*.

## Results

- Surface characterization

**Figure 1:**
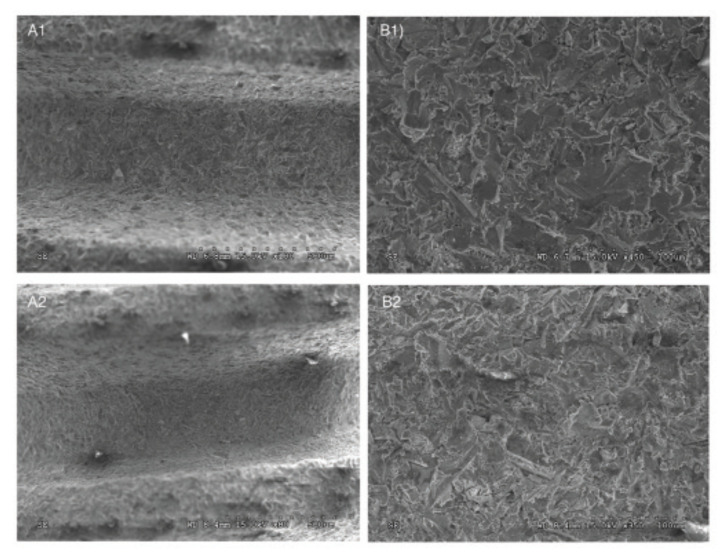
SEM images of both implant designs, D1 and D2 (A and B, respectively), at low and high magnifications (1 and 2, respectively). Similar surface features are observed with no significant differences between groups.

**Figure 2:**
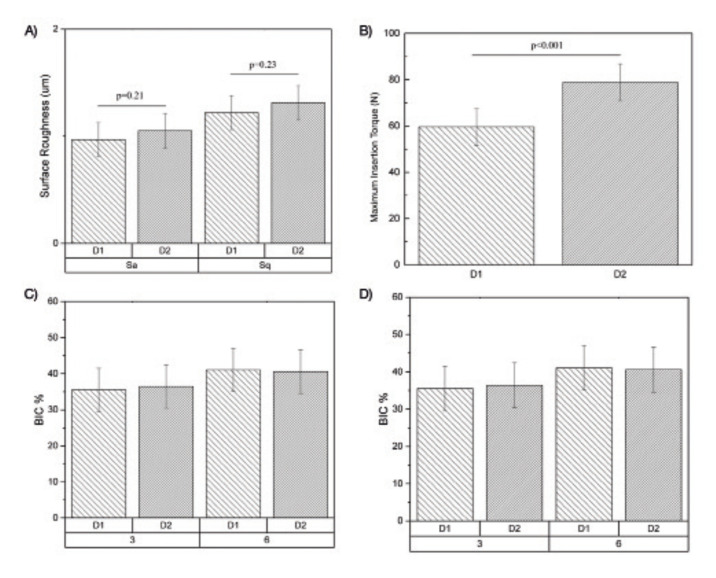
A) Main values and 95% CI of surface roughness parameters (Sa and Sq) of both experimental groups. B) Main values and 95% CI of Maximum Insertion Torque of both experimental groups. Mean values and 95% CI of histomorphometric analyses through C) Bone to Implant contact (%BIC) and D) Bone Area Fraction Occupancy (%BAFO) for both experimental groups at 3 and 6 weeks.

SEM analysis at different magnifications and 15 kV voltage acceleration evidenced similar features in surface detail between implant designs (Fig. 1). This was corroborated quantitatively by surface roughness evaluation using IFM, where Sa and Sq values demonstrated no significant differences (*p*>0.21) between experimental groups (Sa: D1=0.97 ± 0.19 µm, D2=1.05 ± 0.19 µm; Sq: D1=1.21 ± 0.22 µm, D2=1.31 ± 0.22 µm) (Fig. 2).

- Insertion torque, histomorphometric and histologic evaluations

For quantitative measurement of implant primary stability, insertion torque values were significantly lower for D1 regarding the D2 group (59.6 N⋅cm ± 11.1 and 78.9 N⋅cm ± 10.1, respectively; *p*<0.001) (Fig. 2). During the span of the study, no animals were lost or evidenced signs of infection. At the time of euthanasia, no gross inflammatory response was observed, regardless of the implant design, and no implants were lost due to clinical instability.

Histomorphometric evaluations demonstrated no statistically significant differences in BIC (Fig. 2) or BAFO (Fig. 2) results between thread profiles at any time point evaluation (*p*>0.802). While a significant increase in BAFO was observed between 3- and 6-week for both groups (*p*<0.018), no significant differences in BIC were observed between evaluation time points (*p*>0.159).

**Figure 3:**
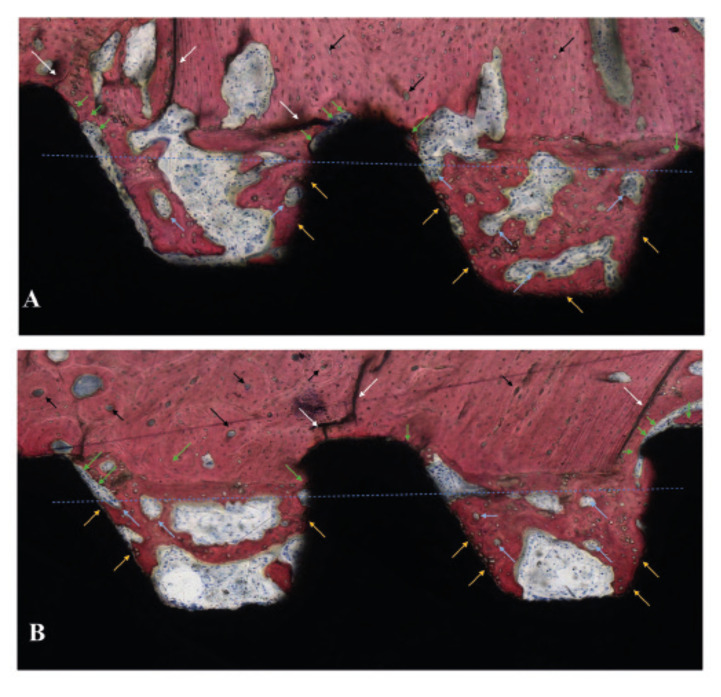
Histology sections of (A) D1 and (B) D2 at 3 weeks. The blue line represents the theoretic position of the osteotomy outer diameter resulting in the formation of healing chambers between the implant inner diameter, the implant threads, and the osteotomy walls. Interfacial remodeling, depicted by green arrows, and bone microcracking, depicted with white arrows, can be observed at the tip of the implant threads where the initial mechanical interlocking took place upon implant insertion. Woven bone formation is observed to occur within the healing chambers from the surgically prepared native bone, from the implant surface (yellow arrows), and from the central region of the chambers, where bone remodeling sites can be observed (clear blue arrows). In the native bone, cortical shell osteonic structures are depicted with black arrows.

**Figure 4:**
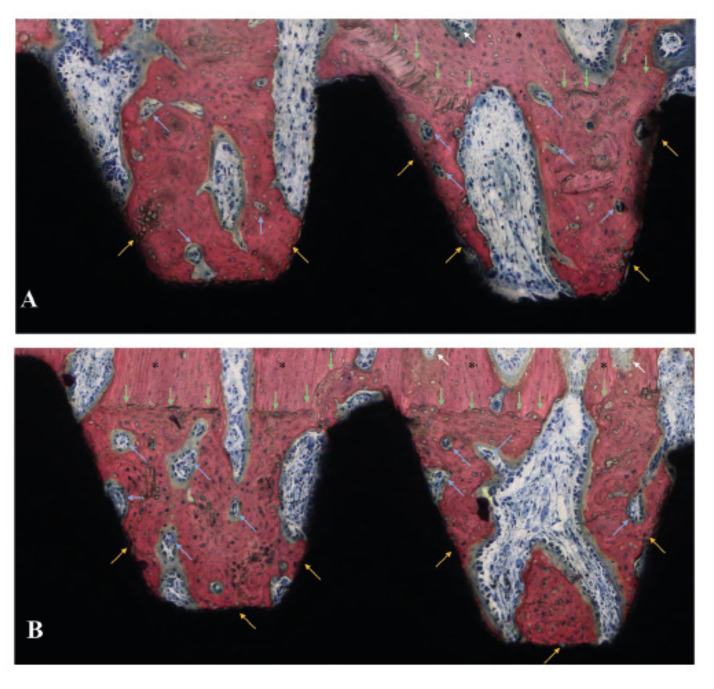
Histology sections of (A) D1 and (B) D2 at 6 weeks. Interfacial remodeling is observed for both groups at the areas of initial bone-implant mechanical interaction, as well as in the outer portion of the healing chambers due to the trauma generated by the osteotomy preparation (green arrows). Within the healing chambers, bone remodeling sites (blue arrows) starting to replace woven bone by mature lamellar bone can be observed. Bone formation from the implant surface (yellow arrows) was observed in similar patterns for both experimental groups. Native bone and bone remodeling sites occurring far from the bone-implant interface are depicted with black asterisks and white arrows, respectively.

Histologic observations were in agreement with the histomorphometric evaluations and are presented in Fig. 3 and Fig. 4. Overall, similar histological features were observed for both groups, and bone formation in direct contact with the implant was observed independent of thread profile and time point evaluation. At 3 weeks, the histological evaluation allowed for the observation of healing chambers formed through the interplay between the osteotomy preparation and the implant macrogeometry. For both groups, a hybrid healing osseointegration pathway was observed, where cortical bone remodeling and bone microcracking due to initial compression at implant placement was observed on the native bone in direct contact with the tips of the implant’s threads. Furthermore, woven bone formation within the healing chamber followed an intramembranous-like healing pattern, where bone growth occurred from the instrumented native bone walls toward the central region of the healing chamber, from the implant surface, and from the center of the healing chamber itself as depicted in Fig. 3.

At 6 weeks, interfacial remodeling was observed for both groups at the areas of initial bone-implant mechanical interaction, as well as in the outer portion of the healing chambers due to the trauma generated by the osteotomy preparation. Within the healing chambers, bone remodeling sites starting to replace woven bone by mature lamellar bone were observed regardless of implant thread design. No immunogenic response cells were identified, and osteoblast activity was readily visible with osteoid formation and vascularity present in the healing chambers (Fig. 4). Over time, a significant increase of woven bone formation within the healing chambers and its subsequent maturation to a lamellar conFiguration was observed from 3- to 6-weeks with similar patterns for both experimental groups.

## Discussion

The successful osseointegration of endosteal implants is dependent on a variety of factors, including the macro-geometric design parameters, which can be modified during manufacturing. The aim of this study was to isolate the effect of variations in geometric thread profile on initial stability and osseointegration parameters of titanium implants with healing chambers in a canine model. Our findings suggest that the increase of the implant contact area, produced by the thread design of D2, significantly increased the maximum insertion torque values with no detrimental effect on the osseointegration process. Therefore, the hypothesis of the present study was accepted.

Macrogeometric implant design has been considered of paramount importance in the achievement of primary stability (6), where excessive micromotion at the bone and implant interface negatively influence osseointegration, possibly leading to implant failure (11,19-21). In the present study, maximum insertion torque evaluation depicted significantly higher values for D2, which had a 17% larger outer surface thread area than D1. Due to this increased thread area in direct contact with the osteotomy walls, forces may have been dissipated over a wider area along the cutting edge of the implant and allowed for greater bone-implant surface interaction and higher initial implant stability (22). Although the reduction of micromotion is desirable, it should not be at the expense of excessive stress on bone (7,23,24). It has been suggested that thicker implant threads may produce excessive bone compression and detrimentally affect osseointegration (25). However, the similar histological patterns observed for both experimental groups in the present study suggest no deleterious bone response after the implantation of devices designed with progressive thicker threads and healing chambers. Such conFiguration seems to support bone healing with the additional advantage of providing high torque values during insertion. Furthermore, the interplay of implant macrogeometry and surgical technique for implant placement was tailored to avoid a purely interfacial osseointegration pathway (16). Qualitative histological evaluation suggest that the healing chambers were filled with the blood clot and evolved towards an osteogenic connective tissue that subsequently ossified through an intramembranous-like pathway, as the wound healing sequence previously described for implants with healing chambers (5,26), where no evidence of bone dieback was observed for D1 or D2.

Quantitative histomorphometric results, BIC and BAFO, for 3- and 6-weeks did not yield significant differences when comparing thread patterns. A statically significant increase of BAFO was observed between 3- and 6-weeks, with no significant differences on BIC. These results suggest that primary stability can be increased via geometric thread profile modifications without detrimental effects on the achievement of secondary stability after implantation. Moreover, the steady increase in BAFO suggest that differences in thread profiles did not cause a significant difference in the histological response at the bone/implant interface over time with a significant increase in bone formation and maturation within the healing chambers over the course of 6-weeks, irrespective of the thread profile.

The surface roughness evaluation demonstrated Sa and Sq values similar to those previously described in the literature for Ti-6Al-4V Grade 5 surfaces treated by micro blasting (27), where Sa was found to be in the limit between minimally and moderately rough surfaces (~1 µm) (28). The absence of differences in surface roughness between D1 and D2 confirmed the isolation of macrogeometric design as the unique difference between both groups. While the results of the present study are encouraging with respect to potential avenues to improve initial implant stability without risking excessive bone resorption, further investigations including a greater range of thread profile modifications, surgical preparations and evaluation time points are warranted to better elucidate the ideal thread design to optimize implant stability and bone response at implant placement.

## Conclusions

Within the limitations of this animal study, both thread profiles had equal potential to successfully osseointegrate into the radial diaphysis of beagle dogs, with D2 having the additional benefit of providing higher mechanical retention upon placement without detrimental bone resorption.
